# Multimorbidity in rheumatic conditions

**DOI:** 10.1007/s00508-016-1090-x

**Published:** 2016-10-13

**Authors:** Helga Radner

**Affiliations:** Department of Internal Medicine III, Division of Rheumatology, Medical University Vienna, Waehringer Guertel 18–20, Vienna, 1090 Austria

**Keywords:** Multimorbidity, Quality of Life, Comorbidity, Rheumatic conditions, Rheumatoid Arthritis

## Abstract

In recent years, multimorbidity in rheumatic conditions has gained increasing attention. Rheumatologist care for an aging patient population with multiple diseases, therefore multimorbidity is the rule, not the exception. Owing to the high prevalence and the potential interaction of coexisting diseases, multimorbidity needs to be taken into account when treating patients with chronic inflammatory conditions. In this review we address the most prevalent comorbidities in patients with rheumatic conditions and their impact on important outcomes, such as physical function, quality of life, and mortality.

Over the last decade, interest in the concept of comorbidity and multimorbidity has increased [1–3]. This is likely due to the fact that clinicians are facing an aging population with multiple morbid conditions occurring in one individual. Especially for the rheumatologist, treating mainly chronic systemic inflammatory diseases, multimorbidity is the rule not the exception. The average rheumatoid arthritis (RA) patient has 1.6 additional conditions, increasing with age, disease duration, and/or disease activity.

Multimorbidity is defined as the “co-existence of two or more chronic diseases in the same individual.” It is a holistic concept, taking into account all potential interactions of co-existing diseases and its impact on the patient’s overall well-being [4]. For rheumatologists, understanding the complex role of multimorbidity is indispensable to provide safe, efficient, and optimal care of our patients.

## Prevalence of multimorbidity in rheumatic conditions

Based on the published literature, the prevalence of multimorbidity in the general population is about 25 % [1], but prevalence estimates vary widely depending on the cohort’s age distribution and methods used to assess multimorbidity. The systemic inflammatory pathophysiological component of rheumatic conditions is inevitably accompanied by multiple other conditions in one individual and, therefore, multimorbid patients are highly prevalent in rheumatology [5]. Approximately two third of patients with RA are considered to be multimorbid. Studies have shown that certain morbidities co-exist because of a shared risk factor profile leading to high co-occurrence rates, such as smoking, obesity, or a sedentary lifestyle. Some studies describe an association of multimorbidity with female gender and low socioeconomic status, but only a few studies have investigated the causes and risk factors for multimorbid patients in detail [1, 6]. Data on prevalence rates of multimorbidity mainly exist for RA patients, whereas for other rheumatic conditions such as psoriatic arthritis (PsA), systemic lupus erythematosus (SLE), or ankylosing spondylitis (AS), only limited data are available. Also for other chronic inflammatory conditions such as inflammatory bowel disease (IBD), only a few studies are available addressing the incidence and importance of multimorbidity [7]. The highest prevalence rates of cardiovascular diseases, depression, and osteoporosis can be observed in RA patients [8, 9]. The prevalence rates of the main conditions for distinct rheumatic conditions are listed in Table 1.

**Table 1:** Prevalence of different morbid conditions in inflammatory rheumatic conditions

Morbid conditions	RA (%) [8, 50]	SpA (%) [51]	SLE (%) [18, 52–56]
*Cardiovascular disease*	6	3	6–10
*Cardiovascular risk factors*			
Hypertension Dyslipidemia Diabetes	40 32 14	34 27 9	40 36–60 11
*Osteoporosis*	30	13	23
*Cancer (any solid)*	5	3	3.2
*Depression*	15	11	46

*RA* rheumatoid arthritis, *SLE* systemic lupus erythematosus, *SpA* spondylarthropathies

### Malignancies

Rheumatic patients are at increased risk of developing certain malignancies, mainly lymphoproliferative disorders in RA, SLE, or Sjoegren syndrome [10, 11]. For other malignancies such as colorectal cancer, a decreased risk can be observed, possibly due to chronic use of NSAIDs.

Over the last few years, increasing evidence was found demonstrating there is no increased risk of most cancers for RA patients on tumor necrosis factor (TNF)-inhibitor therapy compared with conventional disease-modifying anti-rheumatic drug (DMARD) therapy such as methotrexate or leflunomide, and that the risk of cancer did not increase over time for patients on TNF inhibitor therapy [12]. Furthermore, in retrospective case control studies, the risk of cancer recurrence in RA patients treated with TNF inhibitors was similar to that for TNF-naïve patients [13, 14].

### Cardiovascular disease

Owing to the chronic inflammatory character, a shared risk profile such as smoking or physical inactivity and a higher risk of cardiovascular (CV) morbidity and mortality can be observed in many rheumatic conditions such as RA or PsA [15–18]. Young women with SLE may be up to 50 times more likely to suffer from myocardial infarction compared with population-based controls [19]. The rate of CVD in patients with AS is not fully clear with some studies showing a higher prevalence compared with the general population [20, 21], losing statistical significance after adjusting for NSAID use [22]. The stringent control of disease activity plays a central role in minimizing CV risk as it leads to a reduction of inflammation. It has been shown that despite disease control, DMARDs can also improve lipid profile [23, 24] or diabetes [25], which reduces the risk of CV outcomes. The European League Against Rheumatism (EULAR) has acknowledged the importance of CV disease in inflammatory arthritis and has provided recommendations for CV risk assessment and management [26].

### Osteoporosis and pulmonary disease

A higher prevalence of osteoporosis can be observed in patients with rheumatic conditions. The increased risk is mainly induced by glucocorticoid therapy but is also due to immobility and inflammation [27]. Especially in SLE patients, an increased incidence of osteoporosis and symptomatic fractures can be observed, which is mainly related to steroid therapy, disease duration, and disease severity [28]. The increased risk of major fracture in RA patients is taken into account in various osteoporosis risk assessment tools [29].

Recent studies demonstrated an increased prevalence and risk of mortality due to respiratory disease in RA patients independent of smoking [30].

## Impact of multimorbidity on important outcomes

Owing the increasing prevalence of multimorbidity, the clinical but also health economic implications have become more prominent, with an exponential rise in costs with increasing number of concomitant chronic conditions [31]. Multimorbidity can impact therapeutic decisions as well as important clinical outcomes.

### Treatment

It is known from previous studies that multimorbid patients with RA are potentially undertreated for their concomitant disease [32], which might have a negative effect on RA disease activity. Furthermore, we were able to show different treatment patterns in multimorbid RA patients, with a significant lower odds of biological DMARD use in multimorbid RA patients, even if needed because of persisting high disease activity. This is of special importance as it was shown that a more intensive treatment of rheumatic conditions leads to a significant improvement of co-existing conditions, such as CV disease or metabolic syndrome [33, 34].

Guidelines such as “treat to target” or EULAR treatment recommendations were developed in order to achieve optimal therapeutic outcomes in patients with rheumatic conditions. Such strategies based on systematic literature reviews and expert opinion were developed for patients with a single rheumatic condition only and therefore might overlook multimorbidity [35]. Owing to the given inclusion and exclusion criteria, clinical trials exclude multimorbid patients, which limits the generalizability of results and therefore makes it difficult to apply recommendations to daily routine patients. Further studies are needed to assess the effectiveness and safety of DMARD therapy in the subgroup of multimorbid rheumatic patients.

### Disease activity

It was shown that with an increasing number of co-existing morbidities per patients, a higher activity of the rheumatic condition could be observed. Multimorbid patients are less likely to achieve treatment targets such as remission or low disease activity. We found that even after adjusting for important covariates such as age, disease duration, or number of previous DMARDs, the odds of achieving remission decreased by 28 % per additional morbidity 1 year after DMARD initiation [36–38]. This might be due to the interaction of co-existing diseases, polypharmacy, or the tendency to undertreat multimorbid patients [39].

**Fig. 1:**
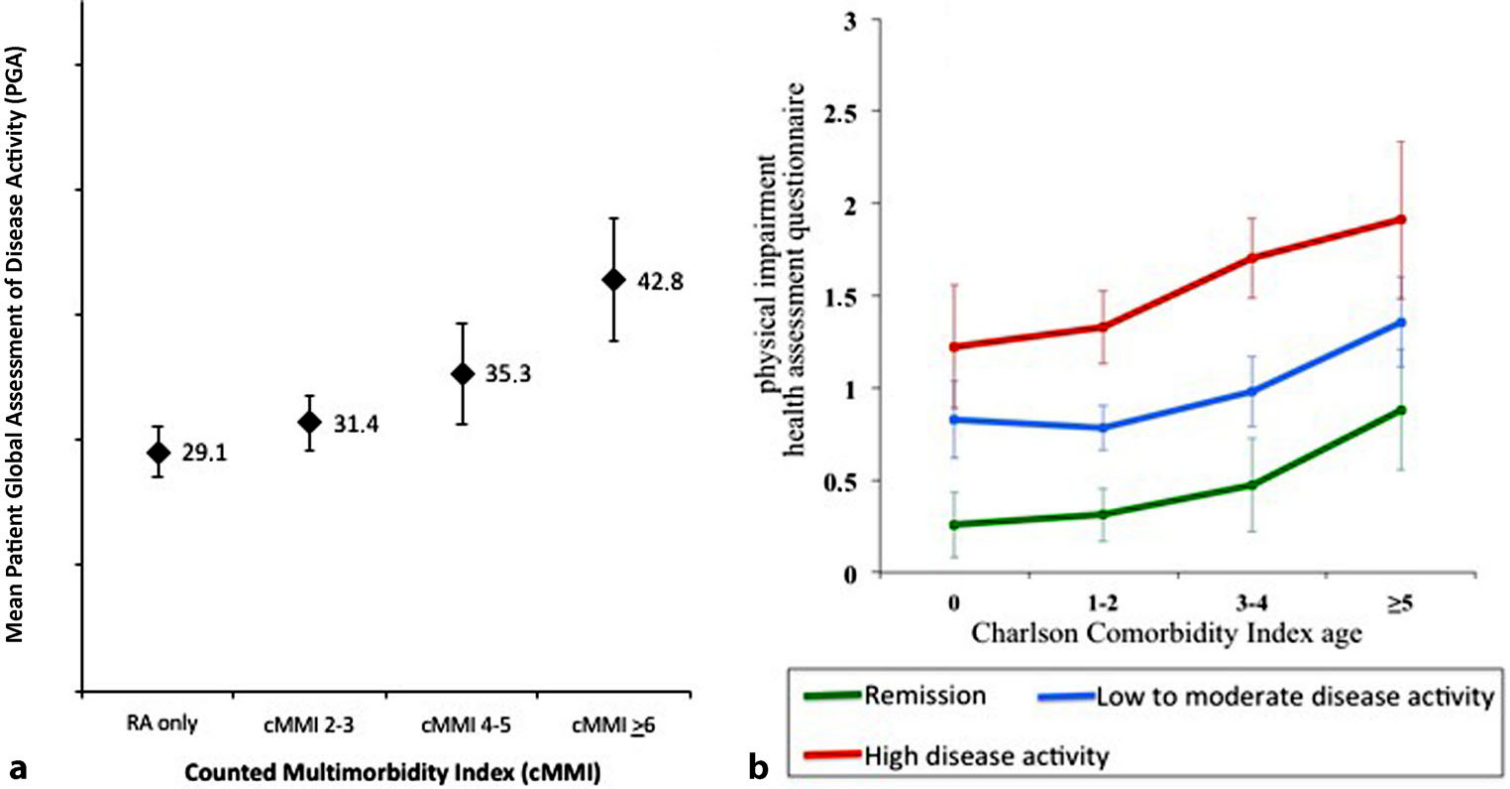
Impact of multimorbidity on disease-related outcomes: **a** increase of patients’ global assessment of disease activity with rising number of morbid conditions per patients; **b** physical impairment increasing with increasing number of morbidities

Furthermore, measures of disease activity developed to assess treatment targets, such as the disease activity score (DAS) or the clinical disease activity index (CDAI), comprise patient-reported outcomes such as patient global assessment of disease activity (PGA), which might be directly affected by multimorbidity. In a recent study of RA patients we found an almost linear increase of PGA with increasing number of morbidities per patient, independent of RA disease activity (Fig. 1a; [40]).

### Physical function and quality of life

The burden of multimorbidity has a negative impact on important outcomes such as health-related quality of life (HRQoL) or physical function, independent of disease activity. Even for RA patients in remission, a significant increase of physical impairment with an increasing number of concomitant diseases was found (Fig. 1b), underlining the overall burden of multimorbidity not only for the individual patient but also for society because of higher rates of work disability [41–43]. In population-based studies it was shown that the negative impact of multimorbidity on function and HRQol is highest in patients having a rheumatic condition as part of their multimorbidity [44].

### Mortality

Over the last decade after introducing biological DMARD therapy, an improvement in disease activity and increasing remission rates were achieved. Nevertheless, despite optimum treatment strategies, a so-called mortality gap can be observed, showing a decrease in mortality in the general population whereas mortality rates in patients with rheumatic conditions remain high or even show a trend of increasing over time [45]. Radovits et al. showed that concomitant conditions are the strongest predictor of mortality [46]. In register studies of RA patients, cardiovascular and respiratory mortality were increased [47, 48]; for other rheumatic conditions, higher mortality due to cancer was found [49].

## Conclusion

In patients with chronic inflammatory rheumatic conditions, multimorbidity is highly prevalent with an impact on important clinical outcomes such as disease activity, physical function, or mortality. By understanding and implementing the concept of multimorbidity, quality of care and clinical outcomes can be improved.

## References

[1] Barnett K, Mercer SW, Norbury M (2012). Epidemiology of multimorbidity and implications for health care, research, and medical education: a cross-sectional study. Lancet.

[2] Diederichs C, Berger K, Bartels DB (2010). The measurement of multiple chronic diseases – a systematic review on existing multimorbidity indices. J Gerontol A Biol Sci Med Sci.

[3] Fortin M, Stewart M, Poitras ME (2012). A systematic review of prevalence studies on multimorbidity: toward a more uniform methodology. Ann Fam Med.

[4] Radner H, Yoshida K, Smolen JS (2014). Multimorbidity and rheumatic conditions-enhancing the concept of comorbidity. Nat Rev Rheumatol.

[5] Sattar N, McInnes IB (2005). Vascular comorbidity in rheumatoid arthritis: potential mechanisms and solutions. Curr Opin Rheumatol.

[6] Uijen AA, van de Lisdonk EH (2008). Multimorbidity in primary care: prevalence and trend over the last 20 years. Eur J Gen Pract.

[7] Roman AL, Munoz F (2011). Comorbidity in inflammatory bowel disease. World J Gastroenterol.

[8] Dougados M, Soubrier M, Antunez A (2014). Prevalence of comorbidities in rheumatoid arthritis and evaluation of their monitoring: results of an international, cross-sectional study (COMORA). Ann Rheum Dis.

[9] Gron KL, Ornbjerg LM, Hetland ML (2014). The association of fatigue, comorbidity burden, disease activity, disability and gross domestic product in patients with rheumatoid arthritis. Results from 34 countries participating in the Quest-RA program. Clin Exp Rheumatol.

[10] Bernatsky S, Boivin JF, Joseph L (2005). An international cohort study of cancer in systemic lupus erythematosus. Arthritis Rheum.

[11] Smitten AL, Simon TA, Hochberg MC (2008). A meta-analysis of the incidence of malignancy in adult patients with rheumatoid arthritis. Arthritis Res Ther.

[12] Askling J, van Vollenhoven RF, Granath F (2009). Cancer risk in patients with rheumatoid arthritis treated with anti-tumor necrosis factor alpha therapies: does the risk change with the time since start of treatment?. Arthritis Rheum.

[13] Raaschou P, Frisell T, Askling J (2015). TNF inhibitor therapy and risk of breast cancer recurrence in patients with rheumatoid arthritis: a nationwide cohort study. Ann Rheum Dis.

[14] Strangfeld A, Hierse F, Rau R (2010). Risk of incident or recurrent malignancies among patients with rheumatoid arthritis exposed to biologic therapy in the German biologics register RABBIT. Arthritis Res Ther.

[15] Jamnitski A, Symmons D, Peters MJ (2013). Cardiovascular comorbidities in patients with psoriatic arthritis: a systematic review. Ann Rheum Dis.

[16] Raterman HG, Levels H, Voskuyl AE (2013). HDL protein composition alters from proatherogenic into less atherogenic and proinflammatory in rheumatoid arthritis patients responding to rituximab. Ann Rheum Dis.

[17] Solomon DH, Curhan GC, Rimm EB (2004). Cardiovascular risk factors in women with and without rheumatoid arthritis. Arthritis Rheum.

[18] Murray SG, Yazdany J, Kaiser R (2012). Cardiovascular disease and cognitive dysfunction in systemic lupus erythematosus. Arthritis Care Res (Hoboken).

[19] Manzi S, Meilahn EN, Rairie JE (1997). Age-specific incidence rates of myocardial infarction and angina in women with systemic lupus erythematosus: comparison with the Framingham Study. Am J Epidemiol.

[20] Nielen MM, van Sijl AM, Peters MJ (2012). Cardiovascular disease prevalence in patients with inflammatory arthritis, diabetes mellitus and osteoarthritis: a cross-sectional study in primary care. Bmc Musculoskelet Disord.

[21] Nurmohamed MT, van der Horst-Bruinsma I, Maksymowych WP (2012). Cardiovascular and cerebrovascular diseases in ankylosing spondylitis: current insights. Curr Rheumatol Rep.

[22] Essers I, Stolwijk C, Boonen A (2016). Ankylosing spondylitis and risk of ischaemic heart disease: a population-based cohort study. Ann Rheum Dis.

[23] Morris SJ, Wasko MC, Antohe JL (2011). Hydroxychloroquine use associated with improvement in lipid profiles in rheumatoid arthritis patients. Arthritis Care Res (Hoboken).

[24] Jamnitski A, Visman IM, Peters MJ (2010). Beneficial effect of 1‑year etanercept treatment on the lipid profile in responding patients with rheumatoid arthritis: the ETRA study. Ann Rheum Dis.

[25] Solomon DH, Massarotti E, Garg R (2011). Association between disease-modifying antirheumatic drugs and diabetes risk in patients with rheumatoid arthritis and psoriasis. JAMA.

[26] Peters MJ, Symmons DP, McCarey D (2010). EULAR evidence-based recommendations for cardiovascular risk management in patients with rheumatoid arthritis and other forms of inflammatory arthritis. Ann Rheum Dis.

[27] van Staa TP, Geusens P, Bijlsma JW (2006). Clinical assessment of the long-term risk of fracture in patients with rheumatoid arthritis. Arthritis Rheum.

[28] Bultink IE, Lems WF (2016). Lupus and fractures. Curr Opin Rheumatol.

[29] Kanis JA, Oden A, Johansson H (2009). FRAX and its applications to clinical practice. Bone.

[30] Sparks JA, Chang SC, Liao KP (2015). Rheumatoid arthritis and mortality among women during 36 years of prospective follow-up: results from the Nurses’ Health Study. Arthritis Care Res (Hoboken).

[31] Wolff JL, Starfield B, Anderson G (2002). Prevalence, expenditures, and complications of multiple chronic conditions in the elderly. Arch Intern Med.

[32] Toms TE, Panoulas VF, Douglas KM (2010). Statin use in rheumatoid arthritis in relation to actual cardiovascular risk: evidence for substantial undertreatment of lipid-associated cardiovascular risk?. Ann Rheum Dis.

[33] Costa L, Caso F, Atteno M (2014). Impact of 24-month treatment with etanercept, adalimumab, or methotrexate on metabolic syndrome components in a cohort of 210 psoriatic arthritis patients. Clin Rheumatol.

[34] Dixon WG, Watson KD, Lunt M (2007). Reduction in the incidence of myocardial infarction in patients with rheumatoid arthritis who respond to anti-tumor necrosis factor alpha therapy: results from the British Society for Rheumatology Biologics Register. Arthritis Rheum.

[35] Boyd CM, Darer J, Boult C (2005). Clinical practice guidelines and quality of care for older patients with multiple comorbid diseases: implications for pay for performance. JAMA.

[36] Ranganath VK, Maranian P, Elashoff DA (2013). Comorbidities are associated with poorer outcomes in community patients with rheumatoid arthritis. Rheumatology (Oxford).

[37] Radner H, Yoshida K, Frits M (2015). The impact of multimorbidity status on treatment response in rheumatoid arthritis patients initiating disease-modifying anti-rheumatic drugs. Rheumatology (Oxford).

[38] Eder L, Thavaneswaran A, Chandran V (2015). Obesity is associated with a lower probability of achieving sustained minimal disease activity state among patients with psoriatic arthritis. Ann Rheum Dis.

[39] Radner H, Yoshida K, Hmamouchi I (2015). Treatment patterns of multimorbid patients with rheumatoid arthritis: results from an international cross-sectional study. J Rheumatol.

[40] Radner H, Yoshida K, Tedeschi SK, Frits M, Iannacone C, Shadick N, Weinblatt M, Aletaha D, Smolen JS, Solomon DH (2015). Different perception of disease activity in Multimorbid rheumatoid arthritis patients [abstract]. Arthritis Rheum.

[41] Rupp I, Boshuizen HC, Jacobi CE (2004). Comorbidity in patients with rheumatoid arthritis: effect on health-related quality of life. J Rheumatol.

[42] Radner H, Smolen JS, Aletaha D (2010). Comorbidity affects all domains of physical function and quality of life in patients with rheumatoid arthritis. Rheumatology (Oxford).

[43] Radner H, Smolen JS, Aletaha D (2009). Impact of comorbidity on physical function in patients with rheumatoid arthritis. Ann Rheum Dis.

[44] Loza E, Jover JA, Rodriguez L (2009). Multimorbidity: prevalence, effect on quality of life and daily functioning, and variation of this effect when one condition is a rheumatic disease. Semin Arthritis Rheum.

[45] Gabriel SE, Michaud K (2009). Epidemiological studies in incidence, prevalence, mortality, and comorbidity of the rheumatic diseases. Arthritis Res Ther.

[46] Radovits BJ, Fransen J, Al Shamma S (2010). Excess mortality emerges after 10 years in an inception cohort of early rheumatoid arthritis. Arthritis Care Res (Hoboken).

[47] England BR, Sayles H, Michaud K (2016). Cause-specific mortality in male US veterans with rheumatoid arthritis. Arthritis Care Res (Hoboken).

[48] Sparks JA, Chang SC, Liao KP (2016). Rheumatoid arthritis and mortality among women during 36 years of prospective follow-up: results from the nurses’ health study. Arthritis Care Res (Hoboken).

[49] Mok CC, Kwok CL, Ho LY (2011). Life expectancy, standardized mortality ratios, and causes of death in six rheumatic diseases in Hong Kong, China. Arthritis Rheum.

[50] Hauser B, Riches PL, Wilson JF (2014). Prevalence and clinical prediction of osteoporosis in a contemporary cohort of patients with rheumatoid arthritis. Rheumatology (Oxford).

[51] Molto A, Etcheto A, van der Heijde D (2016). Prevalence of comorbidities and evaluation of their screening in spondyloarthritis: results of the international cross-sectional ASAS-COMOSPA study. Ann Rheum Dis.

[52] Abu-Shakra M, Gladman DD, Urowitz MB (1996). Malignancy in systemic lupus erythematosus. Arthritis Rheum.

[53] Almehed K, Forsblad d’Elia H, Kvist G (2007). Prevalence and risk factors of osteoporosis in female SLE patients-extended report. Rheumatology (Oxford).

[54] Nery FG, Borba EF, Viana VS (2008). Prevalence of depressive and anxiety disorders in systemic lupus erythematosus and their association with anti-ribosomal P antibodies. Prog Neuropsychopharmacol Biol Psychiatry.

[55] Sabio JM, Vargas-Hitos JA, Navarrete-Navarrete N (2011). Prevalence of and factors associated with hypertension in young and old women with systemic lupus erythematosus. J Rheumatol.

[56] Tselios K, Koumaras C, Gladman DD (2016). Dyslipidemia in systemic lupus erythematosus: just another comorbidity?. Semin Arthritis Rheum.

